# Temporal-Spatial Redundancy Reduction in Video Sequences: A Motion-Based Entropy-Driven Attention Approach

**DOI:** 10.3390/biomimetics10040192

**Published:** 2025-03-21

**Authors:** Ye Yuan, Baolei Wu, Zifan Mo, Weiye Liu, Ji Hong, Zongdao Li, Jian Liu, Na Liu

**Affiliations:** 1 Institute of Machine Intelligence, University of Shanghai for Science and Technology, Shanghai 200093, China; yuanye_usst@usst.edu.cn (Y.Y.);; 2 School of Automation and Electronic Information, Xiangtan University, Xiangtan 411105, China

**Keywords:** human visual inspiration, motion modeling, motion enhancement, spatio-temporal information entropy, keyframe sampling

## Abstract

The existence of redundant video frames results in a substantial waste of computational resources during video-understanding tasks. Frame sampling is a crucial technique in improving resource utilization. However, existing sampling strategies typically adopt fixed-frame selection, which lacks flexibility in handling different action categories. In this paper, inspired by the neural mechanism of the human visual pathway, we propose an effective and interpretable frame-sampling method called Entropy-Guided Motion Enhancement Sampling (EGMESampler), which can remove redundant spatio-temporal information in videos. Our fundamental motivation is that motion information is an important signal that drives us to adaptively select frames from videos. Thus, we first perform motion modeling in EGMESampler to extract motion information from irrelevant backgrounds. Then, we design an entropy-based dynamic sampling strategy based on motion information to ensure that the sampled frames can cover important information in videos. Finally, we perform attention operations on the motion information and sampled frames to enhance the motion expression of the sampled frames and remove redundant spatial background information. Our EGMESampler can be embedded in existing video processing algorithms, and experiments on five benchmark datasets demonstrate its effectiveness compared to previous fixed-sampling strategies, as well as its generalizability across different video models and datasets.

## 1. Introduction

The volume of video data has increased in recent years, driven by the rapid growth of the mobile network and the widespread use of social media, posing significant challenges for video analysis and processing tasks. For action recognition in videos, deep convolutional networks are widely used due to their efficient processing capabilities regarding video information. Deep convolutional networks are able not only to analyze the content of each frame in a video but also to mine clues from the timing information between video frames. However, this advantage in feature extraction makes deep convolutional networks computationally costly, limiting their widespread applicability. Therefore, improving the training efficiency of deep convolutional networks has become a central topic that researchers are actively addressing.

Video data, unlike static images, contain an additional time dimension, allowing them to convey more information while simultaneously having greater redundancy. Specifically, video information has temporal locality (i.e., the similarity between different time instances) and spatial locality (i.e., the similarity between different scenes). This redundant information is the essential reason for the waste of model computing resources. Therefore, eliminating redundant information is an important way to improve deep-learning models in video-understanding tasks. Frame sampling of video data is a common method to eliminate redundant information. However, the existing sampling strategy usually adopts fixed-frame selection, which lacks flexibility when dealing with different action categories.

**Figure 1:**
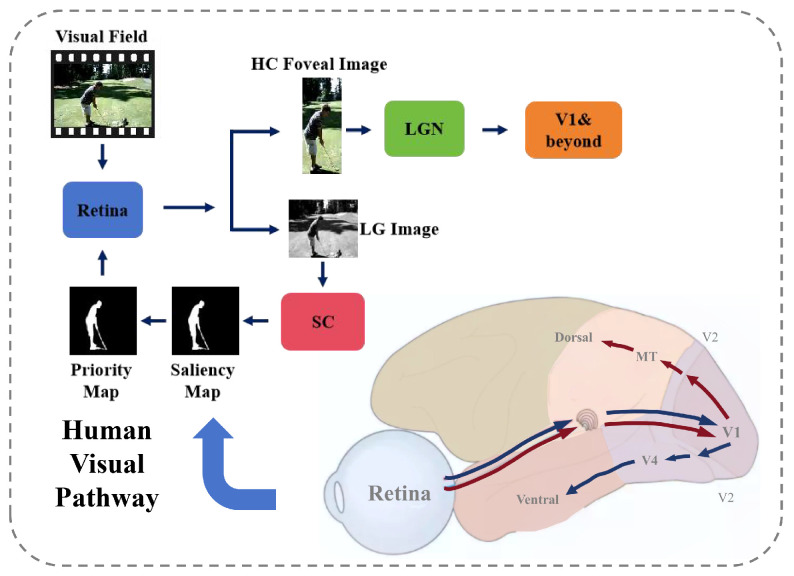
Selective attention in the human visual system. The image is first projected to the retina, which then categorizes the image information and transmits it to different visual pathways. Most color information is transmitted to the LGN, while information that is more colorless is transmitted to the hypothalamus, where a salience map is generated. LGN denotes the Lateral Geniculate Nucleus; SC denotes the Superior Colliculus; V1, V2, V4, and MT represent different regions of the primary visual cortex in the brain.

More and more studies have revealed that the human visual system has an attention mechanism. Specifically, when watching a video, the human eye tends to focus its attention on the most salient target area, rather than evenly distributing it across the entire visual field [1,2,3]. As shown in Figure 1, upon receiving visual signals, the retina transmits the information along two distinct pathways: one pathway relays the signals through the Lateral Geniculate Nucleus (LGN) to the primary visual cortex (V1) and other higher-order visual areas, such as visual cortex V2, V4, and MT, where more complex visual information, including shape, color, and motion, is processed. The other pathway directs the signals to the Superior Colliculus (SC), which plays a key role in modulating visual reflexes and eye movements, facilitating rapid localization and response to visual stimuli. During this process, a new visual scene image is projected onto the retina, and this cycle is repeated iteratively, allowing for continuous attention shifts and refinement.

Grounded in the theory of visual attention, we aspire to develop a model capable of effectively selecting regions that require processing while disregarding non-salient areas, thus reducing computational load and enhancing computational efficiency. In this paper, we propose a simple, efficient, and interpretable operation to eliminate redundant information that does not rely on training data, demonstrating strong generalization capabilities and adaptability to a wide range of video content. Research indicates that object motion tends to evoke high levels of visual attention [4], and embedded motion information serves as a crucial cue for our adaptive selection of frames within a video; entropy is used here as a measure of the richness of motion information. In essence, motion-related frames should possess high motion salience, conveying more motion-related information. Using such prior knowledge about motion, we analyze the importance of video frames and design keyframes based on their significance. Through these keyframes, we perform adaptive sampling that captures most of the motion information, thereby discarding redundant frames with less evident motion information. Subsequently, using motion cues, we highlight the moving parts in the video, guiding the model to selectively attend to the motion components of the video.

Specifically, to implement our proposed operation, we introduce three key components: motion modeling [5], entropy-guided sampling [6], and selective enhancement of the motion of interest [7]. For motion modeling, we used temporal differences at various levels to approximate human motion information. These temporal differences are highly correlated with motion information, providing a degree of indication about the magnitude of motion. Regarding entropy-guided sampling, based on the distribution of motion over time, we propose an entropy-based sampling strategy. Entropy, which represents the degree of disorder in an image, is utilized here to quantify the richness of motion information. We employ entropy values to select frames with significant motion information. Finally, in terms of selective enhancement of the motion of interest, we harness the results of motion modeling as a guide. By performing attention operations between the modeled motion and the original video frames, we emphasize the moving parts while suppressing stationary ones, effectively achieving the suppression of spatial redundancy in the frames. In this way, our model focuses on and efficiently processes the motion-relevant parts of the video, thereby reducing computational costs and increasing overall efficiency.

In summary, our contributions are threefold: (1) We propose an entropy-based dynamic sampling approach that can be embedded into downstream action recognition tasks, significantly reducing frame redundancy. This sampling method enables us to capture the most informative frames, thus improving the efficiency and accuracy of action recognition. (2) We utilize motion cues to improve moving parts in the original frames, effectively reducing redundant spatial information. By emphasizing the motion-related regions, we can improve the representation power of the frames, making them more suitable for action recognition tasks. (3) We conduct extensive experiments on the UCF101 [8], HMDB51 [9], Jester [10], Diving-48 [11], and Sth-Sth V2 [12] datasets, achieving significant improvements over baseline methods. In particular, the adoption of our sampling strategy does not increase the training burden, which makes it highly applicable and practical. These improvements demonstrate the effectiveness of our entropy-guided sampling approach in enhancing action recognition performance.

## 2. Related Work

### 2.1. Video Understanding

The primary goal of video understanding is to recognize human actions in videos. The development of deep learning and the availability of large-scale datasets have made this research more intense. There are two commonly used action recognition solutions: (1) A dual-stream network containing two branches is used to extract spatial and motion information from the data, and then the two types of information are fused to identify categories of actions. TS-CNN [13] is commonly used for dual-stream models. Numerous other methods have been proposed using this design [14]. (2) A 2D convolutional network is used to extract frame-level features from each frame, followed by a feature filtering method to obtain video-level features from frame-level representations, such as NetVLAD [15] and NetFV [16]. Finally, action recognition is performed based on the video-level features. Recently, researchers have become interested in using 3D convolution to recognize video actions. Due to the low-rank approximation, 3D convolution offers low computing complexity while still allowing for the extraction of temporal relations from video data. Some 3D convolutional networks have been designed for video action recognition, such as P3D [17], R(2+1)D [18], and S3D [19]. However, eliminating redundant information in videos to further reduce computational consumption is still a long-term research topic.

### 2.2. Frame Sampling

Frame sampling is a crucial step in optimizing resource efficiency in video-understanding tasks. Some simple yet effective frame-sampling methods are proposed, based on the assumption that all video frames are equal [20,21]. However, these imprecise approaches will inevitably cause the loss of crucial information. To solve these problems, a significant amount of research currently focuses on selecting significant frames for segment extraction, such as SCSampler [22], TSN [23], and MARL [24]. The SCSampler method first employs a lightweight network to estimate the significant score of each fixed-length segment before sampling [22]. TSN (Temporal Segment Network) is a deep-learning framework for action recognition that models long-term temporal dependencies by sparsely sampling and aggregating multiple video segments [25]. The MARL (Multi-agent Reinforcement Learning) method utilizes multiple agents to select frames in parallel, running multiple iterations through a CNN to generate the final decision for all agents [26].

It can be found that these methods enhance the accuracy of keyframe detection by training additional deep networks to assign importance scores to video frames. However, the introduction of deep networks inevitably increases the computational complexity and training time of these methods. In contrast, our goal is to provide a simple, efficient, and interpretable frame-sampling module without any learning strategies.

### 2.3. Attention Mechanism

Attention mechanisms are inspired by human cognitive processes that can improve model performance by dynamically allocating resources to relevant features. They have been widely applied in video understanding. For example, SENet (Squeeze-and-Excitation Networks) [27,28] establishes interdependencies among channels to adaptively recalibrate channel features. CBAM (Convolutional Block Attention Module) [29,30] combines spatial and channel attention for action recognition in video-understanding tasks. In addition, attention mechanisms have also been used for discriminative feature learning. For example, STC (Spatial-Temporal Channel) [31,32] employs fully connected layers to generate channel and spatial attention for 3D convolution, designing enhanced branches at the channel level where the same attention weights are shared across all frames. TEA (Temporal Excitation and Aggregation) [33,34] utilizes feature differences between adjacent frames to generate weights, with the aim of highlighting motion information in videos using a limited temporal receptive field. Inspired by the attention mechanism, we hope to endow the model the ability to distinguish salient motion information from non-salient information, thereby eliminating non-salient redundant information.

## 3. Methods

In this section, we will provide a detailed description of our EGMESampler module. Firstly, we outline the overall framework of the EGMESampler. Then, we introduce the specifics of modeling motion information. Subsequently, we elaborate on the concept of using entropy to guide frame sampling. Finally, we delve into the details of the attention mechanism in our model.

### 3.1. Overview

To efficiently compress videos into clips composed of keyframes while preserving sufficient spatio-temporal cues for video recognition, we propose EGMESampler, an efficient framework for compressing videos into clips. Compared to traditional fixed-sampling methods for action recognition, EGMESampler offers greater flexibility and can significantly reduce computational overhead without sacrificing accuracy. By efficiently extracting and filtering motion information, EGMESampler mitigates the impact of irrelevant backgrounds and enhances the receptive field in the temporal dimension. EGMESampler can be used as a subsequent recognition step for any video recognition network, such as SlowFast [21] and others.

Firstly, we expect the sampler to be able to identify the salience of motion along the temporal dimension based on the richness of motion information, distinguishing frames related to motion from the background. Secondly, we expect the sampler to automatically filter out frames with significant motion based on the distribution of motion information across the temporal dimension and for these frames to represent the entire video action, capturing important details of the motion instances. To achieve these requirements, we adopt frame differencing to capture motion information, striking a balance between accuracy and efficiency. For sampling, we use entropy to dynamically select keyframes, ensuring that the sampled frames cover important motion segments throughout the video. Finally, we perform an attention operation between the extracted motion information features and the keyframes, emphasizing the moving parts of the video. Next, we will describe each of these components in detail.

### 3.2. Overall Architecture

**Figure 2:**
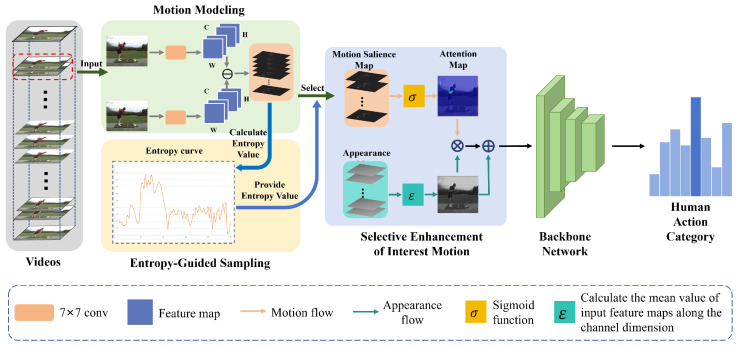
Overall framework of EGMESampler. The framework consists of three components: motion modeling, entropy-guided sampling, and selective enhancement of interest motion. The first component is used to extract appearance cues, the second component is employed to select frames based on entropy values, and the third component is utilized to enhance motion regions within the video. The framework processes the video by convolving adjacent frames to create feature maps and subtracting corresponding channels to generate feature difference maps. It then calculates the entropy of these maps, selects the relevant difference maps and original frames, and applies attention operations. Finally, the motion-enhanced frames are input into video-understanding models for action recognition. Note that the appearance map is actually the selected original image frame in videos.

The overall framework of EGMESampler is illustrated in Figure 2. Given a video sequence, we first complete motion modeling by estimating the variation in image apparent features along the temporal dimension to characterize the object in motion. This enables us to capture the dynamism of motion apparent in videos. Subsequently, we use entropy, which quantifies the uncertainty or unpredictability associated with each frame, to quantify the extracted motion changes. Based on the entropy, keyframes that represent significant motion patterns can be selected. Then, we perform an attention operation with the keyframes to emphasize the moving parts. This attention mechanism allows the model to focus on relevant motion regions, enhancing their importance in the recognition. The resulting enhanced motion video sequence is then fed into the backbone network for recognition. The backbone, which can be any suitable video recognition architecture (such as SlowFast), is trained to extract discriminatory features from the frames and classify the video accordingly. By integrating motion modeling, entropy-based keyframe selection, and attention mechanisms, our approach aims to improve the efficiency and accuracy of video recognition tasks, focusing on the most informative and discriminatory motion patterns present in the video data.

### 3.3. Motion Modeling

To accurately delineate the boundary and texture of the moving target, our approach employs simple convolution networks to extract shallow features from the image, and then the pixel-level difference between the features of successive image frames is calculated. Specifically, given any two adjacent frames, eight 7×7 convolution kernels are applied to the images to obtain shallow feature maps $F_{1}\in R^{H\times W\times C}$ and $F_{2}\in R^{H\times W\times C}$ without reducing spatial resolution. Subsequently, we extend the subtraction to the feature space, as follows:

(1) $$D_{t}^{i}\left(x,y\right)=F_{t}^{i}\left(x,y\right)-F_{t-1}^{i}\left(x,y\right),t\in 2,3,\ldots ,T$$

where i=1,2,3…,C, and *C* is the channel number. $D_{t}^{i}\left(x,y\right)$ represents the pixel-level difference in feature maps between the current frame *t* and the previous frame t−1 at pixel location (x,y). $F_{t}^{i}\left(x,y\right)$ and $F_{t-1}^{i}\left(x,y\right)$ denote the feature maps at the corresponding pixel locations in the current and previous frames, respectively. By computing pixel-level difference in feature space, we can capture more precise motion boundaries and textures than those operating solely on the raw pixel values. To represent differences at the feature level, we aggregate the pixel-level features of *C* channels into a single channel, denoted as $D\in R^{H\times W}$, as follows:

(2) $$D_{t}\left(x,y\right)=\sum_{y=1}^{H}\sum_{x=1}^{W}\sqrt{\sum_{i=1}^{C}D_{t}^{i}{\left(x,y\right)}^{2}},t\in 2,3,\ldots ,T$$

where the aggregation establishes a mapping from appearance to dynamic motion, denoted as $R^{H\times W\times C}\to R^{H\times W}$. The mapping $D_{t}$ effectively encodes the motion signal of an image $F_{t}\left(x,y\right)$, bridging the gap between its appearance and dynamic characteristics.

### 3.4. Entropy-Guided Sampling

Following the acquisition of motion information $D_{t}\left(x,y\right)$, we quantify the motion data using entropy, and then perform adaptive sampling based on entropy value. In information theory, entropy serves as a metric for quantifying the uncertainty and disorder inherent in information. When applied to images, entropy provides insight into the distribution of pixel values and the interrelationship among pixels. Generally speaking, images with higher entropy values exhibit more textures and details, while those with lower entropy values are smoother and have fewer details. To calculate the entropy of images, we encode the motion information $D_{t}\left(x,y\right)$ using a softmax function, resulting in a probability change matrix that represents the variations within the image. Subsequently, we calculate the entropy value of the obtained probability matrix, which measures the amount of information contained in the probability distribution derived from the softmax function, as follows:

(3) $${\mathrm{Entropy}}_{t}=-\sum_{T}\mathrm{Softmax}\left(D_{t}\left(x,y\right)\right)\log_{2}\left(\mathrm{Softmax}\left(D_{t}\left(x,y\right)\right)\right)$$

**Figure 3:**
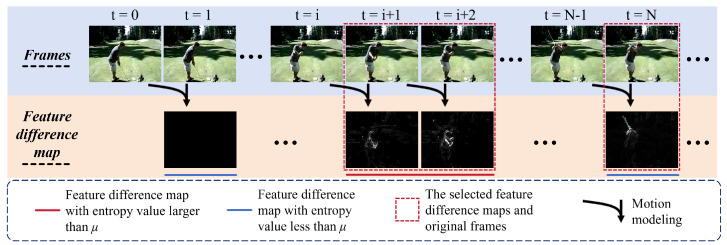
Schematic diagram of entropy-guided sampling. We first obtain the feature difference maps of adjacent frames in videos and calculate their entropy values. The feature difference map and the original frame are then selected based on these entropy values and the threshold μ.

According to the entropy value calculated by the feature difference map, we will set a threshold of $\mu \in \left[0,1\right]$ to judge which of the maps are retained and which of the maps are eliminated. It is worth noting that, when we select the feature difference maps, we will also select the original frames in the video accordingly, as shown in Figure 3. The finally selected feature difference maps and original frames are represented as $p_{m}$ and $f_{a}$, respectively. Unlike fixed sampling, our entropy-guided sampling approach is able to adaptively select frames according to motion intensity, guaranteeing that the sampled frames encompass important motion segments. The sampled frames are then reconstructed into a video sequence and fed into downstream action recognition networks for action recognition tasks.

### 3.5. Selective Enhancement of Interest Motion (SEIM)

To better reduce the redundant information in image space, we use motion information to highlight some important positions or elements in appearance features (i.e., use motion salience to highlight appearance features). Assuming that $p_{m}$ represents the motion salience map (i.e., feature difference map) and $f_{a}$ represents the appearance map (i.e., original image frame in videos), a simple and direct method for reducing the redundant information in image space is as follows:

(4) $$f_{a}^{\prime }=f_{a}⊗p_{m}$$

where $f_{a}^{\prime }$, $f_{a}$, and $p_{m}$ are sizes of C×H×W, C×H×W, and H×W, respectively. ⊗ denotes element-wise multiplication, namely, applying element-wise multiplication between $p_{m}$ and each channel slice of $f_{a}$. However, this has certain limitations. Only part of the moving foreground is in motion, so the stationary part of the moving foreground is 0 in $p_{m}$. Then, simple multiplication cannot ensure a relatively complete motion foreground. Our goal is not to “block out” the non-significant motion regions but to highlight the significant motion regions.

We can articulate this attention by translating the motion salience map into spatial weights, which are subsequently combined with another tensor. By doing so, we can focus on specific regions or elements of the appearance features that are important for motion analysis. This allows us to emphasize significant motion patterns and highlight regions or elements that exhibit significant motion activity, effectively reducing the influence of non-significant motion and noise. The attention operation can be formulated as follows:

(5) $$f_{a}^{\prime }=\varepsilon \left(f_{a}\right)⊗\sigma \left(p_{m}\right)+\varepsilon \left(f_{a}\right)$$

where the attention term $f_{a}$ complements the features that may be incorrectly suppressed by $f_{a}⊗p_{m}$. Thus, the residua formulation is promising to highlight salient-motion parts without discarding still but salient areas. $\sigma \left(\cdot \right)\in R^{H\times W}$ is the sigmoid function, and $\varepsilon \left(\cdot \right)\in R^{H\times W}$ donates calculating the average value of the feature maps of all channel dimensions.

## 4. Experiments

### 4.1. Datasets and Implementations

**Datasets.** Our method was evaluated on six video datasets, which can be divided into two categories. The first category is motion-related datasets, in which action information rather than static information is the key to understanding the action, including Sth-V1 [12], Sth-V2 [12], Diving [11], and Jester [10]. In the Sth-V1 and Sth-V2 datasets, the same action is performed on different objects, which forces the model to understand the basic action rather than identify the object. It includes approximately 100,000 videos across 174 categories. The Jester dataset is a collection of labeled video clips that show humans performing gestures in front of webcams. It contains 148,000 videos across 27 categories. The Diving dataset aims to reduce biases in action recognition due to scene and object context. It has a fine-grained classification and covers 48 different diving types, with a total of 18,000 videos.

The second category type involves scene-related datasets, in which the actions are greatly affected by the context within the scenes, including UCF101 [8] and HMDB51 [9]. The UCF101 dataset is derived from 13,320 manually labeled videos that span 101 distinct action categories. The HMDB51 dataset is compiled from a variety of sources, including videos and movies, and has proven to be both authentic and challenging. It comprises 6766 manually labeled video segments distributed among 51 unique action categories.

**Implementation.** All experiments are conducted on MMAction2 [35], which is an open-source toolkit based onPyTorch v2.4.0 that supports numerous video-understanding models. The models, including TSM [36] and TEA [33], are combined with the entropy-guided sampling strategy to verify our proposed method. These models serve as backbone networks, as shown in Figure 2. During the training, we set the batch size, initial learning rate, weight decay, and dropout rate to 32, 0.01, 0.0005, and 0.5, respectively. We used Stochastic Gradient Descent (SGD) to train the network for 50 epochs, and the learning rate was reduced by 10 times at 20 and 40 epochs. During the testing, we iteratively extracted segments containing *T* frames from the entropy-guided sampled video and used center cropping to adjust the frames to 224×224 for action prediction. The settings of the training and testing phases are consistent on all the datasets. Following [36] and [33], the hyperparameter *T* is set to 8. The experiments used two NVIDIA 2080Ti GPUs.

### 4.2. Experiment Results

**Experiment on entropy-guided sampling.** For entropy-guided sampling, the threshold μ is actually a hyperparameter that determines the sampling rate of video frames, ultimately affecting the performance of the method. A threshold μ equal to 1 indicates that the video frame is not sampled. The threshold μ value is less than 1, indicating that the top percent 100μ of the video frame is selected based on the entropy value, which ranges from high to tiny. To investigate the impact of different values of the hyperparameter μ on the video-understanding task, we conducted a series of ablation experiments using the TSM model [36] as the backbone network, as shown in Table 1. It can be observed that, as the threshold μ increases, the model performance first improves and then declines. The highest performance is achieved when the threshold μ is set to 0.5. This means that half of the frames in a video will be eliminated.

The results indicate that removing 90% of the frames (i.e., μ=0.1) has little impact on human action recognition performance. This is due to the redundancy in video content. When redundancy is high, even a significant reduction in frames does not substantially affect recognition results. This phenomenon occurs because each video clip in the Sth-V2 dataset contains only a single action, leading to inherently high redundancy. Additionally, an interesting phenomenon is observed when the threshold μ is set to 1.0, meaning no frames are removed from the video. In this case, the model’s performance in recognizing human actions actually declines. This suggests that excessive redundancy in the video can also be detrimental to human action recognition.

**Comparison of different methods.** To illustrate the effectiveness of entropy-guided sampling, we compared it with two sampling strategies: (1) fixed stride sampling [14], where fixed strides (s=4) are selected from the video; (2) segment based sampling such as TSN [25] and MGSampler [5], which uniformly sampled fragments along the temporal dimension. We compared the methods based on two criteria: recognition accuracy and processing speed, with the results presented in Table 2 and Table 3. Our proposed method outperforms Fixed Stride Sampling and TSN in terms of recognition accuracy for human action recognition in videos, although it is slightly behind the MGSampler method. To assess the processing speeds, we calculated the average processing speed of each method on the test sets of the UCF101 and HMDB51 datasets. Our method is significantly faster than both TSN and MGSampler. This performance advantage is mainly due to our approach using computationally efficient and interpretable information entropy to assess the importance of video frames, whereas the other two methods rely on complex deep networks to infer frame importance scores.

**Experiment on motion enhancement.** In our method, we introduce a selective motion enhancement technique designed to emphasize the most relevant motion components in videos, with the goal of improving the model’s ability to recognize human actions more effectively. By selectively boosting motion features, we aim to enhance the model’s focus on critical action cues while reducing the influence of irrelevant or static frames. To assess the effectiveness of this enhancement, we conducted experiments across four datasets. As shown in Table 4, the results clearly demonstrate that the model performs better when motion enhancement is applied, compared to when it is not. This improvement suggests that selectively enhancing motion features plays a crucial role in boosting the model’s recognition accuracy, highlighting the importance of motion information for more accurate and robust action recognition.

We also compared the effects of motion enhancement under two conditions, using frame difference and image difference, with the results shown in Table 5. The results indicate that keyframe detection using feature difference outperforms keyframe detection based on image difference, which is consistent with previous research [5]. This can be attributed to the fact that low-level features, such as gradients and textures, are more sensitive to subtle motion variations, particularly at motion boundaries. In contrast, image difference relies on pixel-level changes, which might overlook finer details in motion, especially in complex or subtle action scenarios. Therefore, feature difference provides a more robust and precise approach for keyframe detection, enhancing the effectiveness of motion enhancement in human action recognition tasks.

**Figure 4:**
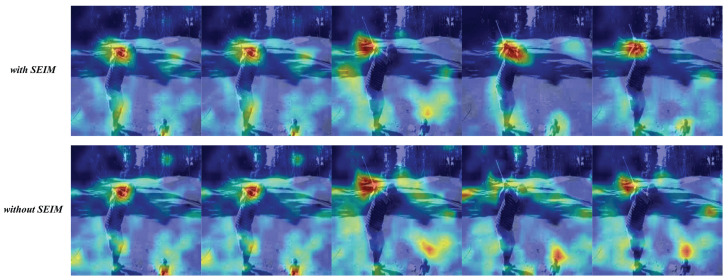
Visualization of the experiments on the motion enhancement. It can be seen that the color of the moving area (hand swing) in the video tends to be red, indicating that it has received greater attention from the model. Moreover, the red areas in our method are more accurate than those in previous methods, which indicates that our method performs better. The TEA model is used to serve as the backbone network.

To gain a deeper understanding of the role of motion enhancement, we visualize the results in Figure 4. Different colors are used to represent the model’s attention to various regions of the image, with red indicating areas that receive more attention. As per our design, the model is intended to focus more on the moving parts of the video while paying less attention to static regions. From Figure 4, we can observe that the hand swing region receives more attention from the model, demonstrating that our method outperforms previous approaches. This improvement is primarily due to the motion enhancement technique, which amplifies the motion components of the video, allowing the model to concentrate on the foreground while minimizing the interference of redundant background information.

**Comparison of different models.** To further validate the effectiveness and generalizability of our method, we integrate our entropy-guided sampling strategy into existing video-understanding models, such as TSM and TEA, as a generic module. This integration allows us to assess the versatility of our approach across different model architectures. We then compare the performance of our enhanced models with previous state-of-the-art methods, with the results presented inTable 6. The results show that our model consistently outperforms all previous methods on both the Sth-V1 and Sth-V2 datasets, with a particularly significant improvement on the Sth-V1 dataset. This demonstrates the robustness and efficacy of our entropy-guided sampling strategy in enhancing model performance. In terms of computational complexity, while our method is slightly more computationally intensive than many previous models, it is still considerably more efficient than video-understanding models that utilize 3D convolutional neural networks. This highlights the balance our approach strikes between performance and computational efficiency, making it a promising solution for large-scale video-understanding tasks.

## 5. Discussion and Conclusions

In this paper, inspired by neural mechanisms of the human visual pathway, we propose EGMESampler, a effective, universal, and interpretable framework designed to eliminate redundant information in temporal and spatial domains. By capitalizing on general prior knowledge regarding motion information, we devise a sampling scheme that is both efficient and effective. Our entropy-guided sampling methodology comprises three integral components: motion modeling, entropy-guided sampling, and selective enhancement of interest motion. This approach ensures that our sampled frames comprehensively encompass all crucial frames exhibiting high motion salience, while emphasizing motion-intensive regions in space. As a result, redundant information is effectively purged from both spatial and temporal perspectives. Extensive experiments conducted on benchmark datasets have conclusively demonstrated the efficacy of our sampling strategy, as well as its robust generalization capabilities across a range of models and datasets. Our proposed method increases the computational and parameter requirements to a certain extent, and our future work aims to reduce the computational and parameter requirements while ensuring model accuracy.

The experimental results demonstrate that our method has broad potential applications in real-world scenarios. For instance, in video compression, it can reduce storage and transmission costs by selectively preserving the most informative frames. In video summarization, it can generate concise yet meaningful highlights by emphasizing key motion-intensive segments. Moreover, in surveillance and action recognition, our approach can enhance anomaly detection and event analysis by focusing on critical movements while filtering out redundant data. These applications highlight the versatility of our method, making it a promising solution for efficient and scalable video processing across various domains.

## Figures and Tables

**Table 1:** The influence of different μ values on the action recognition in the Sth-V2 dataset. The TSM model is used to serve as the backbone network.

μ	0.1	0.2	0.3	0.5	0.7	0.8	1.0
**Top-1**	56.1	57.2	58.6	59.7	59.2	59.4	58.4

**Table 2:** Performance comparison of different methods ^1^.

Method	Top-1
Fixed stride sampling	54.7
TSN	58.4
MGSampler	60.1
**EGMESampler (ours)**	**59.7 **

^1^ The values are obtained from model evaluating on the Sth-V2 dataset (μ=0.5), using TSM as the backbone network.

**Table 3:** Process speed comparison of different methods ^1^.

Method	UCF101	HMDB51
TSN (each video)	6.5 ms	4.7 ms
MGSampler (each videos)	6.9 ms	5.0 ms
**Ours (each video)**	**4.8 ms **	**4.6 ms **

^1^ The values are obtained from model evaluating on the Sth-V2 dataset (μ=0.5), using TSM as the backbone network.

**Table 4:** Comparison of model performance (Top-1) with and without motion enhancement ^1^.

Dataset	EGMSampler (With SEIM)	EGMSampler (Without SEIM)
UCF-101	95.1	94.7
HMDB-51	73.7	72.8
Jester	97.2	96.7
Diving-48	39.4	36.1

^1^ TSM model is used to serve as the backbone network (μ=0.5).

**Table 5:** Comparison between feature difference and image difference ^1^.

Method	UCF-101	Diving-48
Feature difference with SEIM	95.1	39.4
Feature difference without SEIM	94.7	36.1
Image difference with SEIM	94.3	36.6
Image difference without SEIM	93.4	34.9

^1^ TSM model is used to serve as the backbone network (μ=0.5).

**Table 6:** Performance comparison of different models on Sth-V1 and Sth-V2 datasets.

Model	Backbone	Frames	Sth-V1 (Top-1)	Sth-V2 (Top-1)
I3D [14]	3D ResNet50	32 × 3 × 2	41.6	-
NL I3D [37]	3D ResNet50	32 × 3 × 2	44.4	-
TSN [25]	ResNet50	8 × 1 × 1	19.7	27.8
TSM [36]	ResNet50	8 × 1 × 1	20.9	28.1
BQN [38]	ResNet50	8 × 1 × 1	51.7	-
TCM [39]	ResNet50	8 × 1 × 1	48.2	50.8
GST [40]	ResNet50	8 × 1 × 1	45.8	49.2
TIDRL [41]	ResNet50	8 × 1 × 1	49.7	48.8
MVNet [42]	ResNet50	8 × 1 × 1	50.1	50.2
TEA [33]	ResNet50	8 × 1 × 1	48.9	60.9
Ours (TSM)	ResNet50	8 × 1 × 1	58.8	59.7
Ours (TEA)	ResNet50	8 × 1 × 1	55.7	63.8

## Data Availability

The original contributions presented in the study are included in the article; further inquiries can be directed to the corresponding author.
